# Supplementary material for: Atypical Response to Affective Touch in Children with Autism: Multi-Parametric Exploration of the Autonomic System
Source: J Clin Med. 2022 Dec 1;11(23):7146. doi: 10.3390/jcm11237146 (PMC9737198; doi:10.3390/jcm11237146)
Supplement: Supplementary file 1 [file jcm-11-07146-s001.zip › jcm-1955537-supplementary.pdf]

Supplementary Materials

Statistical results for ASD tested vs non-tested groups

We found statistical differences between ASD tested vs non-tested groups for the CARS (U = 33.50, $p < 0.01$, r = 0.67), verbal IQ (U = 20.50, $p < 0.01$, r = 0.73), and SSP 2 (seeking: U = 13, $p < 0.05$, r = 0.69; avoiding: U = 37, $p = 0.74$; sensitivity: U = 17.50, $p < 0.05$, r = 0.58; registration: U = 36, $p = 0.64$). For the SSP2, these differences can be observed in Figure S1. No statistical difference was found in the ADI-R areas (social interaction: U = 93, $p = 0.47$; verbal communication: U = 18.50, $p = 0.64$; non-verbal communication: U = 43.50, $p = 0.53$; repetitive and restricted behaviors: U = 92.50, $p = 0.45$). No statistical difference was found in the ADOS-2 severity score (U = 57, $p = 0.10$).
We performed a Fisher test to check if there was a difference in the number of ASD children tested and non-tested with a total IQ under 70 and we found a statistical difference ($\chi = 6.18$, $p = 0.01$).

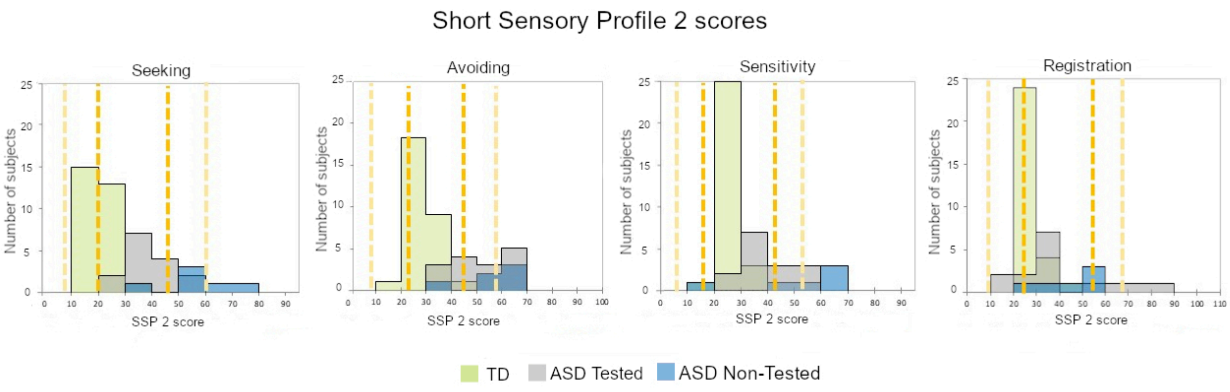

Figure S1: Distribution of SSP2 scores for each area for TD, ASD tested and ASD non-tested groups. The number of participants is represented in bins of 10 points of score in TD children (light green), ASD tested children (grey) and ASD non-tested children (blue). Psychometric references of each area are represented by the dotted bars. The interval between the two dark yellow dotted lines represents the average scores (just like the majority of others = [18-21]-[42-47]). Light yellow dotted lines represent the boundaries with the other ranges: below the average (less than others = [7-8]-[17-20], much less than others = 0-[6-7]) and above the average (more than others [43-48]-[53-60], much more than others = [54-61]-[95-110]).

Correlations between the clinical scores

To evaluate the impact of autism severity on other clinical parameters, we tested possible correlations between the severity of autism scores (ADOS-2 severity and CARS) and the SSP2 scores. No significant correlation was found (Spearman correlations, $p > 0.11$), either for the ASD tested participants, or for the total tested and non-tested ASD children. We also tested correlations between the severity of autism scores (ADOS-2 severity and CARS) and the verbal IQ. We found no correlation for the ADOS-2 severity score ($p > 0.2$, with or without the non-

tested ASD children included). But we found a significant negative correlation between CARS scores and verbal IQ (ASD tested children: $r = -0.63$, $p = 0.037$; ASD tested and non-tested children: $r = -0.65$, $p < 0.001$).

Statistical results for total groups and subgroups comparisons

For the autonomic parameters, the TD_{Pup} group is the only one that differed from the total TD group as only 11 participants were included in order to match the ASD group (maximum amplitude of response: $p < 0.001$; latency of maximum response: $p < 0.001$; AUC: $p = 0.42$; pupil baseline: $p < 0.001$). No difference was found in total TD vs TD_{SCR} ($p > 0.71$), and in total TD vs TD_{RR} ($p > 0.53$).

We found a statistical difference across the four areas of the SSP 2 between TD_{Pup} and ASD_{Pup} (seeking: U = 61, $p < 0.01$, r = 0.67; avoiding: U = 62.5, $p < 0.01$, r = 0.71; sensitivity: U = 62.5, $p < 0.01$, r = 0.71; registration: U = 58.5, $p < 0.01$, r = 0.62), TD_{SCR} and ASD_{SCR} (seeking: U = 262, $p < 0.001$, r = 0.81; avoiding: U = 251, $p < 0.001$, r = 0.75; sensitivity: U = 253, $p < 0.001$, r = 0.76; registration: U = 204, $p < 0.01$, r = 0.45), TD_{RR} and ASD_{RR} (seeking: U =273, $p < 0.001$, r = 0.79; avoiding: U = 265.5, $p < 0.001$, r = 0.75; sensitivity: U = 267, $p < 0.001$, r = 0.76; registration: U = 215, $p < 0.01$, r = 0.45).

We found statistical difference between the TD subgroups and ASD subgroups for the verbal IQ (TD_{Pup} vs ASD_{Pup}: $p < 0.01$, r = 0.80; TD_{SCR} vs ASD_{SCR}: $p < 0.01$, r = 0.79; TD_{RR} vs ASD_{RR}: $p < 0.01$, r = 0.82) but we did no conduct statistical tests on the non-verbal IQ as we have scores for only 4 ASD participants.

No statistical difference in age was found between the TD subgroups and ASD subgroups (TD_{Pup} vs ASD_{Pup}: $t_{(18)} = 1.22$, $p = 0.23$; TD_{SCR} vs ASD_{SCR}: $t_{(34)} = 1.28$, $p = 0.20$; TD_{RR} vs ASD_{RR}: $t_{(36)} = 2.02$, $p = 0.42$) (Table S1).

		pupil	*skin conductance*	*RR interval*	*All*
**TD**	*group name*	TD_{Pup}	TD_{SCR}	TD_{RR}	TD
	n	11	19	20	30
	sex M:F	6:5	8:11	9:11	13:17
	age (± SD)	9.40 ± 1.90	9.56 ± 2.00	9.53 ± 1.82	9.20 ± 0.20
	verbal IQ [range]	127 [99-146]***	125 [99-146]***	123 [88-146]***	124 [88-155]
	non verbal IQ [range]	124 [104-146]*	124 [104-146]*	118 [88-146]*	116 [88-146]
	SSP2:				
	- Avoiding [range]	29 [21-49]**	31 [24-40]***	30 [21-49]***	29 [18-49]
	- Registration [range]	27 [21-37]**	31 [24-40]***	27 [21-41]***	26 [21-41]
	- Sensibility [range]	24 [20-32]**	31 [24-40]***	24 [19-35]***	23 [18-35]
	-Research [range]	21 [15-33]**	21 [15-33]**	21 [15-33]**	20 [13-33]
**ASD**	*group name*	ASD_{Pup}	ASD_{SCR}	ASD_{RR}	ASD
	n	11	19	20	20
	sex M:F	10:1	18:1	19:1	19:1
	age (± SD)	8.54 ± 1.60	9.23 ± 1.71	9.13 ± 1.69	9.13 ± 1.69
	verbal IQ [range]	71 [42-88] n = 5	73 [25-95] n = 13	73 [25-95] n = 13	73 [25-95] n = 13
	non verbal IQ [range]	99 [69-129] n = 2	91 [66-129] n = 4	71 [66-129] n = 4	71 [66-129] n = 4
	CARS [range]	31 [24-40]	30 [24-40] n = 17	30 [24-40] n = 17	30 [24-40] n = 17
	SSP2:				
	- Avoiding [range]	51 [33-63] n = 6	53 [33-68] n = 14	53 [33-68] n = 14	53 [33-68] n = 14
	- Registration [range]	41 [30-80] n = 6	40 [12-80] n = 14	40 [12-80] n = 14	40 [12-80] n = 14
	- Sensibility [range]	38 [26-50] n = 6	40 [26-54] n = 14	40 [26-54] n = 14	40 [26-54] n = 14
	-Research [range]	36 [28-48] n = 6	40 [28-58] n = 14	40 [28-58] n = 14	40 [28-58] n = 14

Table S1: Description of the TD and ASD groups and subgroups. The table reports demographic and clinical data in total TD and ASD groups, and TD and ASD matched subgroups (the total ASD group is the same as the ASD_{RR} group). Values are expressed in mean ± standard deviation (age) and mean with range (minimum and maximum values) for clinical data. The table reports differences for the verbal IQ, non-verbal IQ, and SSP2 four areas between the TD and ASD subgroups. *: $p < 0.05$, **: $p < 0.01$, ***: $p < 0.001$. CARS: Childhood Autism Rating Scale; IQ: Intellectual Quotient; SSP2: Short Sensory Profile 2.
